# Structural validity of the Brazilian version of the Western Ontario and McMaster Universities Osteoarthritis Index among patients with knee osteoarthritis

**DOI:** 10.1590/1516-3180.2020.0046.R1.26062020

**Published:** 2020-10-19

**Authors:** Cheila de Sousa Bacelar Ferreira, Almir Vieira Dibai-Filho, Davi Oliveira da Silva Almeida, Daniela Bassi-Dibai, Felipe Souza Barreto, Adriano Rodrigues de Oliveira, Cid André Fidelis-de-Paula-Gomes

**Affiliations:** I PT. Master's Student, Postgraduate Program on Rehabilitation Sciences, Universidade Nove de Julho (UNINOVE), São Paulo (SP), Brazil.; II PhD. Professor, Postgraduate Program on Physical Education, Universidade Federal do Maranhão (UFMA), São Luís (MA), Brazil.; III Physical Education Teacher and Master's Student, Postgraduate Program on Rehabilitation Sciences, Universidade Nove de Julho (UNINOVE), São Paulo (SP), Brazil.; IV PhD. Professor, Postgraduate Program on Programs Management and Healthcare Services, Universidade Ceuma, São Luís (MA), Brazil.; V PT. Master's Student, Postgraduate Program on Physical Education, Universidade Federal do Maranhão (UFMA), São Luís (MA), Brazil.; VI PhD. Professor, Department of Physical Therapy, Universidade Nove de Julho (UNINOVE), São Paulo (SP), Brazil.; VII PhD. Professor, Postgraduate Program on Rehabilitation Sciences, Universidade Nove de Julho (UNINOVE), São Paulo (SP), Brazil.

**Keywords:** Reproducibility of results, Surveys and questionnaires, Pain, Rheumatology, Joint degeneration, Exploratory factor analysis, Confirmatory factor analysis, WOMAC

## Abstract

**BACKGROUND:**

The original structure of the Western Ontario and McMaster Universities Osteoarthritis Index (WOMAC) has been contested in several languages.

**OBJECTIVE:**

To assess the structural validity of the Brazilian version of WOMAC among patients with knee osteoarthritis.

**DESIGN AND SETTING:**

Structural validity study conducted at physiotherapy clinics and primary healthcare units.

**METHODS:**

The study included males and females aged 40 to 80 years who were all native Brazilian Portuguese speakers, with knee pain in the previous six months and a diagnosis of knee osteoarthritis. We used exploratory factor analysis (EFA) followed by confirmatory factor analysis (CFA) with implementation of a polychoric matrix and the robust diagonally weighted least squares (RDWLS) extraction method. The adequacy of the model was assessed using the following fit indices: root mean square error of approximation (RMSEA), comparative fit index (CFI), Tucker-Lewis index (TLI), standardized root mean square residual (SRMR) and chi-square/degree of freedom (DF).

**RESULTS:**

203 patients with knee osteoarthritis were included. The model proposed in this study with two factors, i.e. “pain” (items 1, 2, 3 and 4) and “physical function” (items 10, 11, 16, 17, 18, 19, 21 and 22), showed adequate fit indices in CFA: chi-square/DF = 1.30; CFI = 0.976; TLI = 0.970; RMSEA = 0.039; and SRMR = 0.070. The factorial loads ranged from 0.68 to 0.76 for the “pain” domain and 0.44 to 0.62 for the “physical function” domain.

**CONCLUSION:**

The Brazilian version of WOMAC with two domains, i.e. “pain” (four items) and “physical function” (eight items), presents the best structure.

## INTRODUCTION

The Western Ontario and McMaster Universities Osteoarthritis Index (WOMAC) is a patient-based self-report instrument that was created and validated in English in 1988, to measure pain, joint stiffness and physical function among patients with hip or knee osteoarthritis. This initial study involved face, content and construct validity, reliability and responsiveness.^1^

Since its creation, WOMAC has been translated, adapted and validated for use in several other languages, such as German, Spanish, Japanese, Swedish and Arabic.^2^ There is also a Brazilian Portuguese language version but, curiously, the study in which the translation, cross-cultural adaptation and validation were performed was not published in a peer-reviewed scientific journal (it was a master's dissertation). However, adequate values for reliability and construct validity were identified.^3^

In that version of WOMAC in Brazilian Portuguese, the structural validity of the questionnaire was not ascertained. In other languages, some studies have investigated the structural validity of WOMAC by means of factor and Rasch analysis. According to a systematic review published in 2015,^2^ factor analysis was conducted in five studies and variation from three to seven in the number of WOMAC domains was observed. Bilbao et al.^4^ highlighted that the Spanish structure of WOMAC with three domains and 24 items was inadequate and proposed a short version with two domains and 11 items, through using confirmatory factor analysis. Rothenfluh et al.^5^ used Rasch analysis and proposed a German version of WOMAC with one domain and 12 items. Also using Rasch analysis, Davis et al.^6^ proposed a new English version of WOMAC with two domains and 17 items.

Thus, considering the different investigations conducted and the different scientific conclusions reached regarding the structure of WOMAC, our study was justified by the need to identify whether the original structure of WOMAC, as used in its translation into the Brazilian Portuguese language, is adequate.

## OBJECTIVE

The aim of this study was to assess the structural validity of the Brazilian version of WOMAC, among patients with knee osteoarthritis.

## METHODS

### Ethical aspects

This study was based on secondary analysis on data from previous studies.^7^^,^^8^ It included participants who had been excluded from these previous studies, but who presented eligibility for the present study. The study procedures were approved by our institution's research ethics committee, under opinion report number 24568013.0.0000.5511, on February 10, 2014. The subjects' participation in the previous studies had been authorized and validated through their signing of an official document.

### Sample

The sample size calculation for the present study was based on the recommendations of the COnsensus-based Standards for the selection of health Measurement INstruments (COSMIN), i.e. seven patients for each questionnaire item, thus resulting in a minimum of 168 patients for WOMAC.^9^

All participants in this study were recruited from the waiting lists of two physiotherapy clinics and five primary healthcare units in the city of São Paulo (SP), Brazil. The study included males and females aged 40 to 80 years who were native Brazilian Portuguese speakers, with knee pain in the previous six months and a diagnosis of knee osteoarthritis based on the criteria established by the American College of Rheumatology, with radiographic confirmation of the diagnosis.^10^ These diagnoses of knee osteoarthritis were made through examination and the written opinion of a physician who was a specialist in rheumatic diseases. The exclusion criteria comprised a history of knee trauma, cognitive impairment, several psychiatric conditions (delirium, neurocognitive disorders or schizophrenia), neurological disorder (sensory or motor) or other disorders of the lower limbs that compromised their functioning.

### WOMAC

This study used the WOMAC version with Likert scale responses. As in the study conducted by Fernandes,^3^ the Brazilian Portuguese version has three domains, namely: “pain” domain with five items (items 1, 2, 3, 4 and 5); “stiffness” domain with two items (items 6 and 7); and “physical function” domain with 17 items (items 8, 9, 10, 11, 12, 13, 14, 15, 16, 17, 18, 19, 20, 21, 22, 23 and 24). For each item, there are five possible answers, ranging from 0 to 4. The score for each domain is calculated as the simple sum of the items answered: in the “pain domain”, the score ranges from 0 to 20; in the “stiffness” domain, the score ranges from 0 to 8; and in the “physical function” domain, the score ranges from 0 to 68 points.

In the original version, the reliability found was considered adequate, with an intraclass coefficient correlation ranging from 0.73 to 0.97. Regarding the construct validity, there was an adequate correlation between the WOMAC domains and the Visual Analogue Scale, Health Assessment Questionnaire and Lequesne Algofunctional Index, with correlation magnitudes ranging from 0.425 to 0.935.

### Statistical analysis

To identify the best WOMAC structure for its version in the Brazilian Portuguese language, exploratory factor analysis (EFA) was initially used, with implementation of a polychoric matrix and the robust diagonally weighted least squares (RDWLS) extraction method, since the response possibilities for each item of WOMAC are ordinal values.^11^^,^^12^ The number of factors to be retained was identified and defined by means of parallel analysis with random permutation of the observed data. The rotation used was robust promin.^13^^,^^14^ Data processing was performed using the free software FACTOR (Universitat Rovira i Virgili, Tarragona, Spain). The adequacy of the model was assessed using Kaiser-Meyer-Olkin (KMO) and Bartlett's test of sphericity. KMO values above 0.70 and significant P-values in Bartlett's test were considered adequate.^15^^,^^16^

Confirmatory factor analysis (CFA) was performed using the R Studio software (Boston, MA, USA) via its lavaan and semPlot packages. The WOMAC questionnaire is scored on a Likert scale (ordinal data). Thus, the CFA was performed with implementation of a polychoric matrix and the RDWLS extraction method, which is more suitable for ordinal variables than the maximum likelihood method.^11^^,^^17^ The model fit was assessed using the following indices: root mean square error of approximation (RMSEA) with 90% confidence interval (CI); comparative fit index (CFI); Tucker-Lewis index (TLI); standardized root mean square residual (SRMR); and chi-square/degree of freedom (DF).

In the present study, values greater than 0.90 were considered adequate according to the CFI and TLI, and values less than 0.08 were considered adequate according to the RMSEA and SRMR. Values below 3.00 were considered adequate in interpreting the chi-square/DF data.^18^^,^^19^ In CFA, factorial loads greater than or equal to 0.40 were considered adequate for the domain. The Akaike information criterion (AIC) and Bayesian information criterion (BIC) were used to compare the models, and the lowest value was considered to be the most appropriate.

## RESULTS

This study included 203 patients with knee osteoarthritis. The personal and clinical characteristics are described in Table 1. In our sample, most of the patients were elderly, female and overweight.

**Table 1:** Characteristics of the study sample (n = 203)

Characteristics		Mean (standard deviation) or n (%)
**Age (years)**		66.89 (4.56)
**Gender**		
	Male	18(8.9%)
	Female	185(91.1%)
**Weight (kg)**		71.53(4.97)
**Height (m)**		1.65 (0.06)
**Body mass index (kg/m^2^)**		26.23 (2.75)
**Numerical pain rating (0-10)**		6.57(1.10)
**WOMAC (2 domains, 12 items)**		
	Pain (0-16)	11.95(1.81)
	Physical function (0-32)	24.18(2.77)

WOMAC = Western Ontario and McMaster Universities Osteoarthritis Index.

**Figure 1:**
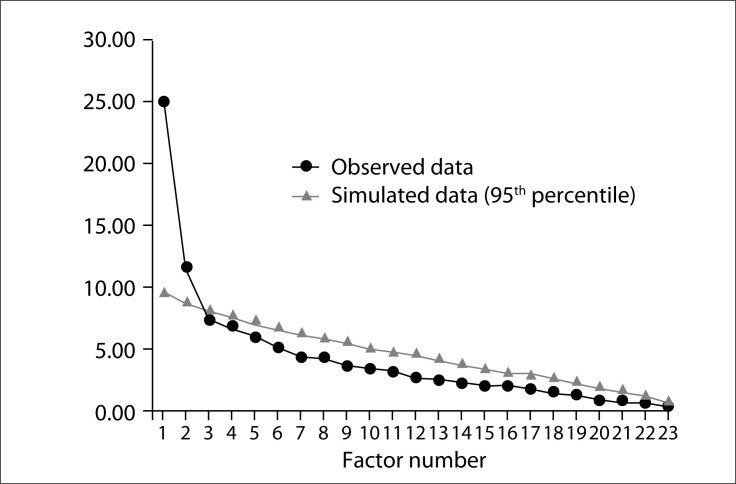
Scree plot from parallel analysis.

The EFA was carried out to explore and identify the structure of the Brazilian version of WOMAC. By means of parallel analysis, two factors were identified: the “pain” domain (items 1 to 5) and the “physical function” domain (items 6 to 24). This WOMAC structure was called Model 1. The EFA with parallel analysis presented suitable fit indices: KMO = 0.75 and Bartlett's test with P < 0.001. Figure 1 presents the scree plot of this parallel analysis with the two factors defined.

From this parallel analysis, we defined Model 2 using the following procedures: items with factorial loads less than 0.50 were excluded (items 6, 7, 8, 9, 12, 13, 14, 15, 20, 23 and 24); item 5 was also excluded because it was originally created for the “pain” domain, but the factorial load of this item allocated it to the “physical function” domain (cross-loading). Therefore, Model 2 was composed of two factors: “pain” (items 1, 2, 3 and 4) and “physical function” (items 10, 11, 16, 17, 18, 19, 21 and 22).

**Figure 2:**
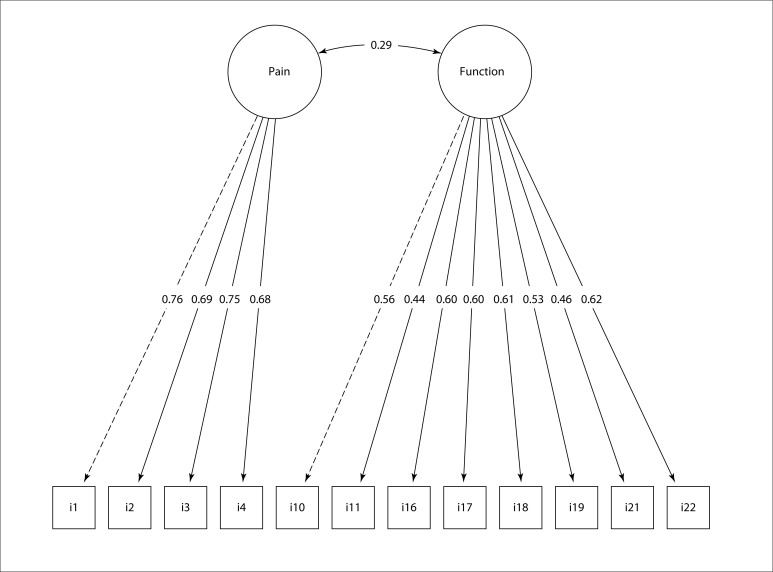
Domains and factorial loads of items of the Western Ontario and McMaster Universities Osteoarthritis Index (WOMAC).

Next, CFA was performed on Model 1 and Model 2. In addition, CFA was performed on the original version conducted by Fernandes^3^ (Model 3) and on the short-form WOMAC proposed by Bilbao et al.^4^ (Model 4). Among these structural models for WOMAC tested here, Model 2 presented the most adequate values for the fit indices and the lowest values of AIC and BIC, as shown in Table 2. The factorial loads for WOMAC with the structure of Model 2 are presented in Figure 2, ranging from 0.68 to 0.76 for the “pain” domain and from 0.44 to 0.62 for the “physical function” domain. The version of WOMAC with the most suitable structure is shown in Appendix 1.

**Table 2:** Confirmatory factor analysis on the four structures of the Western Ontario and McMaster Universities Osteoarthritis Index tested in this study

Models	Chi-square	DF	Chi-square/DF	CFI	TLI	RMSEA (90% CI)	SRMR	AIC	BIC
Model 1	415.205	249	1.66	0.869	0.854	0.057 (0.048-0.067)	0.093	8381.240	8543.587
Model 2	69.288	53	1.30	0.976	0.970	0.039 (0.000-0.063)	0.070	3807.299	3883.503
Model 3	413.064	229	1.80	0.848	0.832	0.063 (0.053-0.073)	0.097	8353.780	8522.754
Model 4	113.145	43	2.63	0.850	0.809	0.090(0.070-0.110)	0.098	4175.547	4258.377

DF = degree of freedom; CFI = comparative fit index; TLI = Tucker-Lewis index; RMSEA = root mean square error of approximation; CI = confidence interval; SRMR: standardized root mean square residual; AIC = Akaike information criterion; BIC: Bayesian information criterion.

Model 1: two domains, four items in the pain domain and 20 items in the physical function domain; Model 2: two domains, four items in the pain domain and eight items in the physical function domain; Model 3: original version proposed by Fernandes, five items in the pain domain, two items in the stiffness domain and 17 items in the physical function domain; Model 4: version proposed by Bilbao et al., three items in the pain domain and eight items in the physical function domain.

## DISCUSSION

Our study revealed that the most suitable structure for WOMAC in Brazilian Portuguese has two domains: four items in the pain domain (items 1, 2, 3 and 4) and eight items in the physical function domain (items 10, 11, 16, 17, 18, 19, 21 and 22). The structural validity of WOMAC has been tested in several studies. According to a systematic review conducted by Gandek,^2^ EFA was carried out on four studies,^20^^–^^23^ and the number of factors (domains) retained for the English and Chinese versions of WOMAC were 3, 4, 5 and 7. This number of domains was higher than the two found in the present study.

We emphasize that our study used parallel analysis as the factor retention method, whereas the abovementioned EFA used other methods for such purposes. Currently, parallel analysis is considered to be a more adequate and robust method for identifying the number of factors in a questionnaire.^24^^,^^25^ Another positive point of the present study is that we implemented factor analysis based on a polychoric matrix and we used RDWLS as an extraction method. These implementations are appropriate and should be used for ordinal categorical data, as in the case of the Likert scale (0, 1, 2, 3 and 4).^11^^,^^12^

In addition to the studies cited in the systematic review conducted by Gandek,^2^ the original WOMAC structure with three domains and 24 items has been rejected by other authors who used the Rasch analysis. Davis et al.^6^ included patients before and after total hip or knee arthroplasty and identified two domains as the best WOMAC structure in English: pain (three items) and physical function (14 items). Another study that investigated the structure of the German version of WOMAC included patients with femoroacetabular impingement and hip osteoarthritis. These authors established a one-dimensional structure for WOMAC with 12 items as the appropriate option.^5^

Bilbao et al.^4^ included patients with hip osteoarthritis and identified a Spanish WOMAC structure with two domains: pain (three items) and physical function (eight items). According to our results, the structure of WOMAC in Brazilian Portuguese also presents the same two domains. However, we identified one more item in the pain domain (item 3), and we also identified eight items in the physical function domain; but of these eight items, only three are in agreement with the aforementioned study (items 10, 16 and 22). In addition, we performed a comparison between different structures for WOMAC; the structure that we proposed presented better fit indices for the model compared with the original structure of WOMAC (three domains; 24 items) and the structure proposed by Bilbao et al.^4^

Our data show that the original version of WOMAC, traditionally used in Brazil by researchers and clinical professionals, should be replaced by the short version presented here. WOMAC is the questionnaire most used to track and identify the signs and symptoms of patients with osteoarthritis. It is an adequate tool for following the clinical changes among patients in the light of therapeutic interventions. We firmly believe that our results, based on factor analysis and model comparison, should serve as a basis for a new understanding of WOMAC and its “pain” and “physical function” domains.

Our study has some limitations. Only structural validity was considered in the present study. Our sample consisted of patients with knee osteoarthritis, and hip osteoarthritis patients were not included. Other important psychometric properties need to be evaluated through future studies, such as reliability and construct validity (correlation with other instruments and questionnaires that measure pain and function).^9^

## CONCLUSION

The Brazilian version of WOMAC with two domains, i.e. “pain” (four items) and “physical function” (eight items), presents the best structure.

## Appendix 1 Short version of Western Ontario and McMaster Universities Osteoarthritis Index (WOMAC) for the Brazilian population.

### DOR

As questões abaixo se referem à intensidade de dor que você geralmente sente devido à artrose. Para cada situação, por favor, marque a intensidade da dor sentida nas últimas 72 horas (marcar suas respostas com um “X”).

#### Qual a intensidade de dor que você apresentou…

1 - Caminhando em um lugar plano?[Table t3]

**Table t3:**

Nenhuma□	Pouca□	Moderada□	Intensa□	Muito Intensa□

2 - Subindo ou descendo escadas?[Table t4]

**Table t4:**

Nenhuma□	Pouca□	Moderada□	Intensa□	Muito Intensa□

3 - Deitado(a) na cama à noite?[Table t5]

**Table t5:**

Nenhuma□	Pouca□	Moderada□	Intensa□	Muito Intensa□

4 - Sentando-se ou deitando-se?[Table t6]

**Table t6:**

Nenhuma□	Pouca□	Moderada□	Intensa□	Muito Intensa□

### FUNÇÃO

As questões abaixo se referem à sua atividade física, isso quer dizer sua capacidade para movimentar-se e para cuidar-se. Para cada uma das atividades abaixo, por favor, marque o grau de dificuldade que você apresentou para realizá-las nas últimas 72 horas devido à artrose (favor marcar suas respostas com um “X”).

#### Qual o grau de dificuldade que você apresentou ao…

10 - Levantar-se estando sentado(a)?[Table t7]

**Table t7:**

Nenhuma□	Pouca□	Moderada□	Intensa□	Muito Intensa□

11 - Ficar em pé?[Table t8]

**Table t8:**

Nenhuma□	Pouca□	Moderada□	Intensa□	Muito Intensa□

16 - Colocar as meias?[Table t9]

**Table t9:**

Nenhuma□	Pouca□	Moderada□	Intensa□	Muito Intensa□

17 - Levantar-se da cama?[Table t10]

**Table t10:**

Nenhuma□	Pouca□	Moderada□	Intensa□	Muito Intensa□

18 - Tirar as meias?[Table t11]

**Table t11:**

Nenhuma□	Pouca□	Moderada□	Intensa□	Muito Intensa□

19 - Ficar deitado(a) na cama?[Table t12]

**Table t12:**

Nenhuma□	Pouca□	Moderada□	Intensa□	Muito Intensa□

21 - Sentar-se?[Table t13]

**Table t13:**

Nenhuma□	Pouca□	Moderada□	Intensa□	Muito Intensa□

22 - Sentar-se ou levantar-se do vaso sanitário?[Table t14]

**Table t14:**

Nenhuma□	Pouca□	Moderada□	Intensa□	Muito Intensa□
